# Bacteriology in Its Relation to Veterinary Science1Read before the Keystone Veterinary Medical Association, Philadelphia, Pa., December 8, 1896.

**Published:** 1897-01

**Authors:** M. P. Ravenal


(To be continued.)

---

### BACTERIOLOGY IN ITS RELATION TO VETERINARY SCIENCE.[1]

By M. P. Ravenal, M.D.

It is with more than usual pleasure that I have accepted your kind invitation to address you this evening, and have chosen as my subject "Bacteriology in its Relation to Veterinary Science."

While the names of Pasteur and Koch will always be the two foremost in this branch of science, the ranks of the veterinarians have furnished a number of men whose work has been epoch-making, and who have added much that is most useful to our knowledge. Nocard, Le Clainche, Chauveau, Strauss, Shutz, Bollinger, Kitt, Johne, Raillet, Peronchito, and Stockflett can never be forgotten, while in the rank and file of veterinary practitioners there have always been a host of earnest and intelligent workers who, though they may not have succeeded in recording brilliant individual achievements, have done noble work and have always given most generous assistance to those who have made a more special study of bacteriology. It is peculiarly fitting, therefore, that veterinarians should take especial interest in the advancement of a science they have done so much to develop.

Each year records new discoveries and opens up possibilities not dreamed of less than a generation ago. The field of preventive medicine has so far proven more productive to the veterinarian than it has to his brothers in the healing art as applied to mankind. Quarantine laws based on bacteriology have as wide a scope or wider for the former as for the latter practitioner. Time does not permit me to go into details which are as well known to you as to

[1] Read before the Keystone Veterinary Medical Association, Philadelphia, Pa., December 8, 1896.

me, and I will only mention briefly the success which has attended preventive inoculations against anthrax, rouget du porc, chicken-cholera, rushbrand, and now known as "vaccines," following the suggestion of Pasteur, who first succeeded in producing an artificial immunity by bacteriological methods and generously proposed the adoption of the general term "vaccines" in honor of Sir Edward Jenner.

Work along these lines is constantly going on in all parts of the world, and so favorable have the results been so far that we have reason to hope that in the near future efficient vaccines will be found against the great majority of diseases to which man and animals are susceptible.

In addition to those just mentioned we now have others for the cure and prevention of tetanus, diphtheria, streptococcus infection, and a certain degree of success has been obtained even against tuberculosis.

Though not strictly germane to my subject, the methods and results are so similar that I may mention also the wonderful results obtained by Calmette and Fraser in the production of immunity against the serpent venoms and the immunizing properties of the blood-serum of the immune animals, called antivenin.

These vaccines are produced by different methods, and the present imperfect understanding we have of susceptibility and immunity makes it impossible so far to have one uniform method of procedure. The nature of the disease also influences us to a certain extent. Thus, while tetanus kills by the extremely virulent toxin formed by the germ, the germ itself never being found far beyond the site of inoculation; anthrax, on the other hand, seems to kill rather by its presence in veritable myriads than by any special poison produced. In the spleen, for instance, the anthrax bacilli are often so numerous as to be more apparent than the tissues of that organ when seen under the microscope. The glomeruli of the kidney are so packed that they frequently burst, and the rods pass into the first part of the tubules. Diphtheria, like tetanus, kills by means of the toxin formed, the germs only rarely being found distributed through the blood or internal organs.

For anthrax and diphtheria we have two distinct types of vaccination, while a third is practised in inoculations against hydrophobia. The success in each has been most marked and abundant. In the first vaccines are obtained by cultivating the anthrax bacillus under conditions which decrease its virulence and make it non-spore bearing. The usual procedure is by cultivation at a temperature somewhat above that at which the germ grows best. According to the length of time to which it is subjected to this treatment, various degrees of virulence are obtained, and these modified cultures are called anthrax vaccines. Two are usually employed, the more attenuated first, followed by the stronger after a few days.

In the second disease—diphtheria—we produce a gradual immunity in an animal, the horse being the one usually selected, by subcutaneous injections of the strongest toxin we can produce by artificial culture of the bacillus, the injections being about eight days apart and the dose being doubled each time, commencing with 1 c.c. and continuing usually until the dose reaches the enormous amount of 200 c.c. During this time what we call antitoxin has been developed in the blood of the animal, and is contained in the serum, which has both therapeutic and prophylactic properties. In anthrax and diphtheria the success attained has been truly wonderful; in tetanus, in which the plan outlined for diphtheria is usually followed, the success has not been great, but through no fault of the method, the chief cause of failure being that treatment is begun too late; for when tetanic symptoms have already shown themselves the cell-destruction has gone so far that repair is impossible in the majority of instances. Professor Roux, of the Pasteur Institute, lays great stress on this point, and with Nocard advises prophylactic rather than therapeutic treatment.

The third type of vaccination, as seen in hydrophobia, consists in producing a rapid immunity by successive inoculations of the virus of the disease, which has been attenuated by exposure to the air and drying, commencing with a feebly virulent injection and ending with a strong one, the treatment being begun after inoculation by the bite of the rabid animal with a supposedly virulent virus.

A fourth type of preventive inoculation is seen in vaccination against smallpox, the occurrence of the mild disease, vaccinia, protecting against the allied but virulent and fatal disease. Not being founded on bacteriological methods, however, it need only be mentioned here in passing.

(To be continued).

---

One hundred sheep inspectors have been appointed in Canada to attest to the freedom from disease of these animals in compliance with the requirements of the United States.

